# Atherosclerosis at your fingertips?

**DOI:** 10.1007/s12471-015-0736-z

**Published:** 2015-07-28

**Authors:** C.A. Swenne

**Affiliations:** Cardiology Department, Leiden University Medical Center, PO Box 9600, 2300 RC Leiden, The Netherlands

The endothelium separates blood and the vascular wall. Endothelial stimulation by agonists or by shear stress has effects on the smooth musculature in the vessel wall by paracrine communication. After the finding that intact endothelium is essential for acetylcholine to produce arterial vasodilation, various agents were discovered that stimulated the endothelium to produce endothelium-derived relaxing or contracting factors (EDRF/EDCF), the major EDRF being nitric oxide (NO). Additionally to vasodilation, NO inhibits platelet and monocyte adhesion and smooth muscle cell proliferation and migration, all key factors in the genesis and progression of atherosclerosis. Thus, dysfunctional endothelium, characterised by limited NO production, has lost part of its vasodilatory function and promotes atherosclerosis.

In 1986, Ludmer and colleagues infused acetylcholine and the NO donor nitro-glycerine in the left anterior descending artery (LAD). Nitro-glycerine invariably caused LAD vasodilation but acetylcholine caused only vasodilation in the normal arteries, while arteries with stenosis showed vasoconstriction or even temporal occlusion. This study paved the road for invasive assessment of coronary endothelial integrity and its association with atherosclerosis in the coronary circulation. In 1989, Cox and colleagues measured flow-mediated LAD diameter changes by quantitative angiography, using adenosine infusions to increase blood flow. Dilation was markedly impaired in atherosclerotic vessels. However, all vessels dilated with nitro-glycerine. Hence, absence of flow-mediated dilation in atherosclerosis reflects impaired endothelial vasodilator function. Later, in 2009, Lavi and colleagues demonstrated that coronary segments with a higher degree of endothelial dysfunction were associated with necrotic plaque.

## Non-invasive flow-mediated dilation and peripheral arterial tonometry

Intracoronary endothelial function assessment, while yielding important information, is not feasible for large-scale application because it is expensive and requires catheterisation. Looking for non-invasive alternatives, measurement of flow-mediated dilation (FMD) of the brachial artery was an important step forward. Measuring in a peripheral arterial bed instead of in the coronary circulation is defendable from the viewpoint that atherosclerosis can be regarded as a systemic condition. FMD is non-invasive: increased flow through the artery is induced by reactive hyperaemia after a 5-minute occlusion of the arm, arterial diameter changes are measured by ultrasound. Peripheral arterial tonometry (PAT) was a next step; this non-invasive method also relies on reactive hyperaemia after a 5-minute occlusion of the arm but compares baseline and reactive hyperaemia plethysmograms measured at a fingertip; hence, PAT is done in a microvascular bed instead of in a conduit artery. Because PAT is low risk, easy to perform and highly operator-independent, it is suitable for large-scale measurement. Consequently, there is much interest to validate this technique in all stages of atherosclerotic disease.

In the current issue of this Journal, Van den Heuvel and colleagues [1] publish a study that assesses the value of PAT to identify subjects at low risk among patients with new onset stable chest pain. Low risk was defined as no revascularisation—PCI or CABG—within one year. In addition to PAT, patients underwent exercise testing, CT angiography and calcium scoring. In contrast to the conventional diagnostics, PAT could not predict low risk.

Earlier, Rubinshtein and colleagues [2] had demonstrated the predictive value of PAT for adverse cardiovascular events (combined endpoint including cardiovascular death, myocardial infarction, revascularisation or cardiac hospitalisation) in a comparable patient group. However, separate prediction of revascularisation could also not be demonstrated. Later, Suessenbacher et al. [3] performed PAT in patients with significant coronary artery disease in at least one coronary vessel plus patients with < 70 % stenosis and a high cardiac risk profile. Average follow-up was 44 months. In this study, PAT did not predict cardiovascular events. Recently, Matsuzawa and colleagues [4] performed PAT in a patient group referred for angiography for chest pain evaluation or abnormal stress test results. PAT results were significantly different in patients with and without significant coronary artery disease.

## Discussion

Negative or mixed conclusions require critical analysis. What can be expected of PAT for the assessment of coronary atherosclerosis? There are several concerns: (1) reactive hyperaemia is not uniquely endothelium dependent; (2) the site of measurement is not representative for the sites where atherosclerotic lesions emerge; (3) the microvasculature in the fingertip abundantly includes anastomoses; (4) endothelial function is subject to many daily-life stimuli/events:

1. The mechanism of reactive hyperaemia is the same as that of autoregulation: with insufficient blood supply, metabolites accumulate in the interstitial space and cause arteriolar vasodilation (smooth muscle cell relaxation) by direct and by endothelium-mediated effects. E.g., smooth muscle cells as well as endothelial cells have adenosine receptors and, consequently, the metabolite adenosine acts directly on smooth muscle but also indirectly, by acting on endothelial cells that in turn produce NO. Hence, microvascular vasodilation does not faithfully represent endothelial function because it is only partly endothelium dependent.
2. Disruption of laminar flow at branching points in the circulation influences the local endothelial layer via mechanotransduction, thus promoting conditions for atherosclerosis; the microvasculature in the fingertips is not representative for such atherosclerosis-prone sites in conduit arteries.
3. Vascular tone in the arteriovenous anastomoses in the finger is sympathetically controlled rather than by NO. This complicates interpretation of PAT measurements in terms of endothelial function.
4. Endothelial function is influenced by daily-life factors such as diet, smoking, hormone level changes during the menstrual cycle, diurnal and seasonal factors, stress, environmental factors like room and skin temperature, exercise, all at the cost of limited reproducibility of PAT measurements.

All mentioned factors weaken the link between PAT measurements and endothelial function, thus hampering reliable atherosclerosis assessment and risk stratification by this technique. Nevertheless, a recent review considering a variety of measures of peripheral artery function [5] gives hope for future clinical use. Possibly, a particular setting (e.g., screening of young asymptomatic persons, risk assessment in chest pain with minimal coronary artery disease, risk assessment in advanced atherosclerosis) requires a particular technique, PAT, FMD or else. Anyway, more research is needed and it will take time before we know if coronary atherosclerosis will be at our fingertips.

### Funding

None.

### Conflict of interests

None declared.
